# Centralisation of services for children with cleft lip or palate in England: a study of hospital episode statistics

**DOI:** 10.1186/1472-6963-12-148

**Published:** 2012-06-10

**Authors:** Kate J Fitzsimons, Shumaila Mukarram, Lynn P Copley, Scott A Deacon, Jan H van der Meulen

**Affiliations:** 1 Clinical Effectiveness Unit, Royal College of Surgeons of England, 35-43 Lincoln’s Inn Fields, London, WC2A 3PE, United Kingdom; 2 South West Cleft Unit, Frenchay Hospital, Bristol, UK; 3 London School of Hygiene and Tropical Medicine, London, UK

## Abstract

**Background:**

In 1998, a process of centralisation was initiated for services for children born with a cleft lip or palate in the UK. We studied the timing of this process in England according to its impact on the number of hospitals and surgeons involved in primary surgical repairs.

**Methods:**

All live born patients with a cleft lip and/or palate born between April 1997 and December 2008 were identified in Hospital Episode Statistics, the database of admissions to English National Health Service hospitals. Children were included if they had diagnostic codes for a cleft as well as procedure codes for a primary surgical cleft repair. Children with codes indicating additional congenital anomalies or syndromes were excluded as their additional problems could have determined when and where they were treated.

**Results:**

We identified 10,892 children with a cleft. 21.0% were excluded because of additional anomalies or syndromes. Of the remaining 8,606 patients, 30.4% had a surgical lip repair only, 41.7% a palate repair only, and 28.0% both a lip and palate repair. The number of hospitals that carried out these primary repairs reduced from 49 in 1997 to 13, with 11 of these performing repairs on at least 40 children born in 2008. The number of surgeons responsible for repairs reduced from 98 to 26, with 22 performing repairs on at least 20 children born in 2008. In the same period, average length of hospital stay reduced from 3.8 to 3.0 days for primary lip repairs, from 3.8 to 3.3 days for primary palate repairs, and from 4.6 to 2.6 days for combined repairs with no evidence for a change in emergency readmission rates. The speed of centralisation varied with the earliest of the nine regions completing it in 2001 and the last in 2007.

**Conclusions:**

Between 1998 and 2007, cleft services in England were centralised. According to a survey among patients’ parents, the quality of cleft care improved in the same period. Surgical care became more consistent with current recommendations. However, key outcomes, including facial appearance and speech, can only be assessed many years after the initial surgical treatment.

## Background

Craniofacial abnormalities are among the most common of all birth defects[1]. In the United Kingdom (UK), about one in 700 live born children has a cleft of the lip or palate or of both[2,3]. The cleft may affect a variety of functions, including speech and hearing. Successful management of patients born with a cleft requires multidisciplinary and highly specialised surgical and non-surgical treatment from birth until adulthood. Primary surgical repair to the lip and palate early in life is critically important for the long-term success of the restoration of facial appearance and function [4].

During the early 1990s, concerns arose about the standards of cleft treatment received by cleft patients in the UK. A multi-centre European study reported that the two participating UK centres were among the weakest on almost every aspect of care assessed [5]. The results showed that 48% of cleft patients treated in these UK centres had dental arch relationships that may have required corrective surgery in late teenage years. The corresponding figure in Norway was 6%.

These findings prompted a national study of care and outcomes in children born with unilateral cleft lip and palate in the UK, conducted in 1996 by the Clinical Standards Advisory Group (CSAG), to investigate the clinical care provided by the National Health Service (NHS) [4]. It showed that outcomes were poor or very poor in 20% of 12 year olds for lip appearance and in 42% for nasal appearance. Almost one fifth of the same age group had speech that was different enough to provoke comment or was unintelligible [6-8].

This study also found that the majority of cleft surgeons in the UK were performing primary repairs on only one patient with unilateral cleft lip and palate per year. The few surgeons performing primary repairs on five or more of these patients per year seemed to have better outcomes in terms of intelligibility of speech, hypernasality, and nasal appearance.

As a result of these findings, CSAG recommended in 1998 that cleft services should be reconfigured into eight to 15 regional specialist cleft units (hospitals providing cleft surgery) with surgeons performing repairs on at least 40 to 50 new cases per year and each hospital treating 100 to 120 new cases per year [4]. In response to these recommendations, the Department of Health initiated the implementation of nine regional “hub-and-spoke” services with one or two specialist cleft units in each region that could provide an array of specialist services, including primary surgical cleft repair [9].

In this paper, we describe the process of centralisation of cleft services in England since 1997. Hospital Episode Statistics (HES) data were used to assess changes in the number of hospitals and surgeons treating cleft patients and in hospital and surgeon volumes. We also establish whether the timing of primary surgical repair and the corresponding lengths of hospital stay and emergency readmission rates have changed.

## Methods

### Data source

Data were extracted from the HES database, which contains records on all admissions to NHS hospitals in England [10]. Diagnostic information is coded using the International Classification of Disease 10^th^ revision (ICD-10), and procedure information is classified according to codes from the Classification of Surgical Operations and Procedures 4^th^ Revision (OPCS-4).

### Patients

Patients born between 1 April 1997 and 31 December 2008 were included if they had at least one HES record of a hospital admission with a diagnosis code for cleft lip and/or palate (ICD-10 codes Q35, Q36 or Q37) as well as a procedure code for a primary cleft repair (OPCS-4 codes F031, F291). All included patients were followed up until 31 December 2009.

Patients were excluded if they had ICD-10 codes for additional congenital anomalies or syndromes as these will have a major impact on when and where they may receive their cleft treatment. A list of the ICD-10 codes used to identify the congenital anomalies and syndromes is available in Additional file 1.

### Hospital and surgeon volume

Hospitals and surgeons performing primary cleft repairs were identified using the NHS provider code and consultant code assigned to the first primary lip or palate repair procedure HES record for each patient. Hospitals and consultants were categorised into patient volume groups, according to patient year of birth. When assessing volume, we only counted the first primary repair procedure in each patient, because treatment protocols differ between hospitals and consultants, particularly with regard to repairing the cleft lip and palate during the same operation or on separate occasions.

### Timing of repair, length of hospital stay and emergency readmissions

The timing of the first primary repair on the lip and the first primary repair on the palate in relation to birth and the mean length of stay in hospital at the time of repair were analysed separately for patients undergoing lip repairs, palate repairs and combined lip and palate repairs. We defined a combined lip and palate repair as a lip repair procedure code and a palate repair procedure code occurring on the same date. In England, of those patients undergoing both a primary lip repair and a primary palate repair, 38% will undergo both repairs at the same time (i.e. combined repair), while 62% have their repairs performed on separate occasions. Length of stay in hospital was defined as the duration between admission and discharge from the hospital performing the primary repair. Emergency readmissions occurring within 48 hours of discharge from hospital were examined to calculate emergency readmission rates. Length of stay was not calculated for children with either a missing admission or discharge date, and children with a missing discharge date were excluded from the readmission analyses.

### Analyses

Data were analysed according to patients’ year of birth. Categorical data are presented as numbers and percentages, and length of stay in hospital is described using means and standard deviations (sd). We used the *χ*^2^ test to assess variations in hospital and consultant volume and analysis of variance to test for changes in length of hospital stay according to year of birth. A p value <0.05 was considered statistically significant. All statistical calculations were performed in Stata 10 (Statacorp, College Station, TX, USA).

## Results

We identified 10,892 patients born between 1 April 1997 and 31 December 2008 with a cleft diagnosis who underwent a surgical primary repair in England by 31 December 2009. Of these, we excluded 2,286 (21%) who had additional congenital anomalies or syndromes. Of the 8,606 non-syndromic cleft patients, 2,615 (30.4%) had a lip repair only, 3,585 (41.7%) had a palate repair only, 925 (10.7%) had a combined lip and palate repair, and 1,481 (17.2%) had a lip and palate repair on separate occasions. The proportion of children with both a cleft lip and cleft palate undergoing combined repairs has fluctuated between 29% and 53% over the years we examined.

### Variation in number of hospitals treating cleft patients

On average, 732 non-syndromic patients underwent a primary surgical cleft repair each year. For children born in 1997 (from 1 April only), primary repairs were performed by 49 hospitals (Table 1). The majority of these hospitals had a low patient volume, with 29 hospitals (59%) treating fewer than 10 new patients per year, and 39 (80%) treating fewer than 20 patients. Only two hospitals treated at least 40 patients. For children born in 1998, 14 (32%) out of 44 hospitals performed repairs on fewer than 10 new patients and 30 (68%) carried out repairs on fewer than 20 patients.

**Table 1:** Number of hospitals in England according to number of patients receiving a primary cleft repair, by patient year of birth

**Year of birth**	**Total number of NHS hospitals**	**Number of patients undergoing primary repairs**				
		**1–4**	**5–9**	**10–19**	**20–39**	**≥40**
Pre-CSAG						
1997^a^	49	12	17	10	8	2
1998	44	7	7	16	12	2
Post-CSAG						
1999	42	6	11	14	6	5
2000	40	10	6	11	7	6
2001	38	10	8	6	9	5
2002	28	8	3	1	10	6
2003	22	5	2	0	8	7
2004	21	4	3	0	6	8
2005	16	1	0	2	4	9
2006	15	2	0	0	4	9
2007	13	0	0	0	1	12
2008	14	1	0	0	2	11

^a^ Births from 1 April to 31 December 2007 only.

The number of hospitals treating non-syndromic cleft patients decreased over time, with all children born in 2007 being treated by 13 hospitals, all of which were designated to become ‘specialist cleft units’ in the wake of the CSAG report. In 2008, one additional hospital, a children’s hospital, performed a primary repair on a child with multiple health problems that were not included in our exclusion criteria for additional congenital abnormalities and syndromes. It should be noted, however, that the consultant operating on the child was a cleft surgeon from the region’s specialist cleft unit.

Table 1 shows that, with the reduction in number of hospitals treating patients with a cleft over this 12-year period, there was an increase in patient volume (p < 0.001). Of the 13 specialist cleft units carrying out cleft surgery on children born in 2007, 12 (92%) performed repairs on at least 40 patients, six (46%) performed them on at least 60, and two (15%) units performed repairs on approximately 100 patients (98 and 101 patients, respectively). For children born in 2008, 11 (85%) of the specialist cleft units performed primary repairs on at least 40 patients and five (39%) performed repairs on at least 60 patients. The highest number of patients undergoing primary repairs in 2008 in one cleft unit was 98.

### Regional variation in the centralisation of services

The 13 specialist cleft units are spread over nine regional networks in England. The speed of cleft service centralisation varied between these regions. The first region completed centralisation in 2001, with all of its non-syndromic cleft patients being treated in the regional specialist cleft unit. A total of four regions had completed the process by 2004, six regions by 2005, and all nine regions by 2007. The speed of centralisation was not determined by the regional proportion of cleft patients who were treated at the time of the CSAG publication by hospitals that were to become specialist cleft units. For example in one region, the future specialist cleft unit treated over 90% of regional cleft patients born at the time of the CSAG publication, but it did not complete centralisation until 2007. In contrast, the hospital that was to become the specialist cleft unit in another region was treating only one third of regional cleft patients born in 1997 but was treating 100% of the patients born in 2003.

### Variation in number of consultants

There were 98 and 102 consultants responsible for primary surgical repair in non-syndromic patients born in 1997 (from 1 April only) and 1998, respectively (Table 2). Over three quarters of these consultants were operating on fewer than 10 new patients per year, with 26 (27%) operating on just one patient born in 1997 and 20 (20%) operating on just one patient born in 1998. Only one consultant had an annual volume of more than 40 patients in both 1997 and 1998 (58 and 78 patients, respectively). The number of consultants performing primary repairs reduced to 24 for children born in 2007 and to 26 for children born in 2008. Over the 12-year period, surgeon volume increased significantly (p < 0.001), with almost two thirds of the consultants each operating on between 20 and 39 children born in 2007 and 2008, and approximately one quarter operating on at least 40 children. The remaining 15% of surgeons had annual volumes of fewer than 20 patients.

**Table 2:** Number of consultants in England according to number of patients receiving a primary cleft repair, by patient year of birth

**Year of birth**	**Total number of consultants**	**Number of patients undergoing primary repairs**				
		**1-4**	**5–9**	**10–19**	**20–39**	**≥40**
Pre-CSAG						
1997^a^	98	62	17	14	4	1
1998	102	53	24	17	7	1
Post-CSAG						
1999	66	25	12	20	6	3
2000	53	18	8	14	8	5
2001	49	16	11	8	11	3
2002	42	16	5	5	11	5
2003	33	9	2	3	16	3
2004	32	6	5	4	15	2
2005	27	4	0	5	15	3
2006	26	5	0	2	14	5
2007	24	1	2	1	14	6
2008	26	3	1	0	16	6

^a^ Births from 1 April to 31 December 2007 only.

Further analyses of the low volume surgeons (those carrying out primary repairs in fewer than 10 patients annually) in 2007 and 2008 indicated that all but one could be explained by either changes in staff circumstances (e.g. retirement, new appointments and cleft services moving away to other hospitals) or coding limitations within HES (i.e. surgeons who had carried out other procedures in the same episode as the primary cleft repair were coded as the responsible consultant for that episode).

### Timing of primary lip and palate repairs

The majority of cleft lips were repaired between three and six months after birth. The proportion of patients receiving their lip repair at this age increased steadily from 47% to 75% over the 12 years of births studied (Table 3). The increased proportion appears mostly to be attributed to the decline in repairs performed very early on in life. For children born in 1997 and 1998, 17% of cleft lips were repaired within the first month of life. This practice ceased in 2004.

**Table 3:** Length of hospital stay and proportion of primary cleft repairs performed at the recommended age

**Year of birth**	**Primary lip repairs**			**Primary palate repairs**		
	**Number of repairs**	Mean (SD) length of stay (days)*	**% performed at age 3–6 months**	**Number of repairs**	**Mean (SD) length of stay (days)**^**§**^	**% performed at age 6–24 months**
Pre-CSAG						
1997-1998^a^	574	3.8 (2.3)	46.9	797	3.8 (2.1)	69.4
Post-CSAG						
1999–2000	669	3.4 (2.5)	49.8	897	3.7 (1.7)	71.2
2001–2002	695	3.3 (2.6)	54.1	866	3.6 (1.8)	74.9
2003–2004	731	3.1 (1.6)	66.4	875	3.4 (2.0)	75.7
2005–2006	683	2.6 (1.4)	75.0	854	3.0 (1.4)	83.5
2007–2008	744	2.0 (1.2)	74.6	777	2.5 (1.4)	86.1
All	4096	3.0 (2.1)	61.8	5066	3.3 (1.8)	76.7

^a^ Births from 1 April 2007 to 31 December 2008 only.

* Mean length of stay for primary lip repairs was calculated for 4087 patients with a known admission and discharge date.

^§^ Mean length of stay for primary palate repairs was calculated for 5037 patients with a known admission and discharge date.

Palate repairs undertaken between 6 months and 2 years of age increased from 69% in 1997 and 1998 to 86% in 2007 and 2008 (Table 3). The main change in timing appears to be a reduction in repairs performed after the age of 2 years. Late repairs (> 2 years) represented 14% of all palate repairs on children born in 1997 and 1998 and only 4% of repairs among those born from 2005 onwards. It should be noted that the figures in the later years are slight underestimates of the number of late repairs, since some children born with a cleft in the latter years of this study may not have been diagnosed within the study period.

Combined lip and palate repairs were most frequently performed between 3 and 6 months of age (results not shown in Table 3). The proportion carried out at this age increased from 57% in 1997 and 1998 to 78% in 2007 and 2008. This increase can be mainly attributed to a reduction in combined repairs performed before 3 months of age.

### Length of stay in hospital

Table 3 also shows a change in the mean length of stay in hospital over the 12 years according to type of cleft repair. The mean number of days spent in hospital decreased steadily for all repair types (p < 0.01) with patients undergoing lip repairs admitted for the shortest duration throughout the period examined. Length of stay for combined lip and palate repairs decreased the most over the 12 years, from 4.6 (sd 1.8) days among those born in 1997 and 1998 to 2.6 (sd 1.9) days for children born in 2007 and 2008. Combined lip and palate procedures represent only 11% of all repairs; these are therefore not shown in Table 3.

### Emergency readmissions

The average rate of emergency readmission after cleft surgery within 48 hours of discharge from hospital over the 12-year period was 1.1% for lip repairs, 0.9% for palate repairs and 1.1% for combined lip and palate repairs. There was no evidence of a trend over time.

## Discussion

### Summary

Since the publication of the recommendations to centralise cleft services in 1998, the number of NHS hospitals involved in providing primary cleft surgery in England has reduced from more than 40 to 13 specialist cleft units in 2008. The time needed to complete the centralisation process varied among the nine regions from two to eight years. As a result, annual hospital volumes have increased considerably with 11 of the 13 specialist cleft units treating more than 40 new non-syndromic patients each year. The number of surgeons responsible for primary cleft repairs reduced in the same period from more than 100 to 26 with 85% of the surgeons treating more than 20 new non-syndromic patients per year.

In the same period, the timing of primary surgical repairs became more consistent with current recommendations (between 3 to 6 months for lip repairs and between 6 to 24 months for palate repairs) and lengths of stay after primary repair decreased from 3.8 to 2.5 days with no effect on emergency readmission rates.

### Methodological considerations

We used HES data, which allowed us to examine care and surgical intervention for every cleft patient admitted to an NHS hospital in England between 1997 and 2009. By doing so, we were able to describe national trends in the provision of cleft surgery over a number of years following the recommendation to centralise cleft services. However, we found several cases where non-cleft surgeons had been reported to HES as the consultant responsible for treating patients undergoing a primary cleft repair. Upon further examination of these cases, it was apparent that these patients received other non-cleft procedures during the same hospital episode.

We recognise that there may be some variation in the way that repair procedures are coded within and between cleft units. For example, it is possible that a primary lip and partial palate repair may be reported using a lip repair code only. However, this does not affect our hospital and surgeon volume analyses, as volume was assessed at a patient level only. Timing of repair and length of stay in hospital may have been influenced by these possible coding limitations, although there is no evidence to suggest there is systematic bias.

The cohort of non-syndromic cleft patients included in our analyses does not represent all patients with a cleft. Syndromic clefts represent approximately 21% of all cleft cases in England. Consequently, the data presented underestimate the total hospital and surgeon cleft caseload by about 27% (= 1/(1–0.21)). As explained earlier, patients with syndromic clefts were excluded as their additional problems could have determined when and where they were treated. As a consequence, the units where these patients receive their treatments do not consistently reflect the configuration of cleft services.

### Centralisation of cleft services

A key recommendation of the CSAG report was that eight to 15 regional units should be created, with each treating 100 to 120 new cleft patients per year, which is equivalent to about 80 to 100 non-syndromic patients [4]. Only two (15%) units in 2007 and three (23%) units in 2008 met the recommended volume. Given the average cleft incidence in England, it could be argued that the original recommended hospital volume was an unrealistic target [11]. However, our analyses reveal that almost all of the 13 specialist cleft units in England treated at least 40 patients in 2007 and 2008, which is comparable with other units in Europe.

Another key CSAG recommendation was that each cleft surgeon should perform primary repairs on at least 40 to 50 new cleft patients a year, which is equivalent to about 30 to 40 for non-syndromic patients. Twelve (50%) out of 24 surgeons in 2007 and eight (31%) out of 26 surgeons in 2008 met this recommendation. Fewer than 5% of surgeons performed repairs on this volume of patients at the time of the CSAG publication, which highlights the progress made in this area over the subsequent 10 years.

The present study found that the timing of primary surgical repairs became more consistent throughout the process of centralisation. In 2004, an international survey on trends in cleft treatment found that 66% of lip repairs were performed between 3 and 6 months of age [12]. We found that in England this proportion was 75% in 2008. This finding is relevant because patients undergoing repairs after four weeks of age have better dental arch relationships than those undergoing repairs in the first four weeks [4]. Also, the timing of primary repairs in England now corresponds more closely to the timings of repairs in some European units that were found by the Eurocleft Study to have some of the best surgical outcomes among cleft patients in Europe[4,13-15].

Length of stay in hospital at the time of primary repair decreased significantly over the 12-year period. A relationship between increasing provider volume and a reduction in hospital stay has been reported by a number of studies [16-18]. A reduction in length of stay has also been observed in other areas of surgery over a similar time period [17]. It is likely that a number of factors have contributed to this reduction. Apart from centralisation, these may include general improvements in technology and surgical techniques, changes in the economic climate requiring improved efficiency and productivity, and the implementation of targets. Although a reduction in length of stay has coincided with the centralisation of cleft services, the nature of our analyses do not allow us to determine a causal relationship.

### Comparison with other clinical areas

Cleft care is not the only area in which centralisation of clinical services has been pursued to improve specialist care for children in the UK. For example, the investigation of paediatric heart surgery at the Bristol Royal Infirmary that commenced in 1998 recommended that this type of surgery should be conducted in fewer and bigger units providing an environment with doctors and nurses trained to look after children [19]. Despite another review that supported the recommendations [20], plans to centralise paediatric cardiac services have not yet been fully implemented and decisions on the geographic configuration of these services in England were still being discussed in 2011 [21].

Another example of reconfiguration is the establishment of regional paediatric intensive care units, initiated by a Department of Health review that was published in 1997 [22]. Progress across the country happened “at variable pace” but it is recognised that considerable progress was made [23]. At the same time, it became clear that concentrating paediatric intensive care affected a number of wider issues, including training and the maintenance of knowledge and skills in dealing with critically ill children in general hospitals and the complexity of organising safe transfer of patients between general hospitals and paediatric intensive care units. It is therefore understandable that the new standards for critically ill children that were published in 2010 aim to consider all disciplines involved in the full patient journey from district general hospitals to paediatric intensive care units.

Both examples of service reconfiguration in England demonstrate that it may take many years to complete the process and that the “blueprint” of the reconfiguration may have to be reconsidered along the way. From this perspective, the implementation of the recommendations of the CSAG report seems rather speedy especially given the complexity of the clinical problem and the number of clinical disciplines involved. Admittedly, our results only chart the reduction in units and clinicians involved in primary surgical repair, but given that the surgery is the starting point of a lifelong treatment programme, it is very likely to reflect the geography of the entire service.

The variable pace at which the reconfiguration of paediatric intensive care took place corresponds to our observation that the centralisation of cleft services was complete in 2001 in the first of the nine regions and only in 2007 in the last. This highlights that specific local circumstances are important factors determining the speed of service reconfiguration.

### Outcomes

A survey carried out in 2007 by the Cleft Lip & Plate Association, a national voluntary organisation of all people with and affected by a cleft lip or palate in the UK, among 227 parents of children with a cleft under the age of three, concluded that cleft services and outcomes had clearly improved [24]. Parents responding to the survey valued the specialist nurses providing early care, the standard of surgery provided by experienced surgeons, and multidisciplinary teams offering parents easier access to the clinicians involved in the treatment of their child. However, parents also highlighted that access to orthodontists, paediatricians and psychologists needed further improvement. The main driver for the recommendation to centralise cleft services was that it would provide better outcomes for patients. There is a rapidly growing body of evidence that, in general, outcomes are better among patients treated in larger units [4,25-27]. There is also evidence that this is the case in the area of cleft care [5,28]. However, key outcomes of cleft care, including dental arch relationships, facial appearance, speech, and hearing, all of which have an important bearing on the quality of life of the cleft patient, are not known until many years after the surgical procedure. As a result, it will take between five to ten more years from now before the actual impact of centralisation of cleft services on these outcomes can be studied and evaluated.

## Conclusions

Between 1998 and 2007, cleft services in England underwent a process of centralisation. There are now significantly fewer hospitals and surgeons performing surgical cleft repairs, and, consequently, cleft hospitals and surgeons have significantly higher patient volumes. Longer term follow-up is needed to determine the impact on cleft outcomes.

## Abbreviations

CSAG: Clinical Standards Advisory Group; HES: Hospital Episode Statistics; ICD: International Classification of Disease; NHS: National Health Service; OPCS: Classification of Surgical Operations and Procedures.

## Competing interests

The authors declare that they have no competing interests.

## Authors’ contributions

KF and SM wrote the first draft of the manuscript. LC acquired the data, and LC and SM performed the analyses. SD edited the clinical content of the manuscript. JvdM conceived of the study, participated in its design and coordination and edited the manuscript. All authors read and approved the final manuscript.

## Funding

This work was funded by the national Specialist Commissioning Group for England and the Wales Specialised Health Services Committee and was carried out by the team of the CRANE Database (the national cleft database for England, Wales and Northern Ireland), which is overseen by the UK NHS Cleft Development Group.

## Pre-publication history

The pre-publication history for this paper can be accessed here:

http://www.biomedcentral.com/1472-6963/12/148/prepub
